# *FOXL2* homozygous genotype and chromosome instability are associated with recurrence in adult granulosa cell tumors of the ovary

**DOI:** 10.18632/oncotarget.27447

**Published:** 2020-01-28

**Authors:** Francois Kraus, Julie Dremaux, Wajd Altakfi, Magalie Goux, Léa Pontois, Henri Sevestre, Stéphanie Trudel

**Affiliations:** ^1^ EA4666, LNPC, Université de Picardie Jules Verne, Amiens, France; ^2^ Laboratoire d’Oncobiologie moléculaire, Centre Hospitalier Universitaire Amiens-Picardie, Amiens, France; ^3^ Service de Gynécologie et Obstétrique, Centre Hospitalier Universitaire Amiens-Picardie, Amiens, France; ^4^ Service d’Anatomie et de Cytologie Pathologiques, Centre Hospitalier Universitaire Amiens-Picardie, Amiens, France

**Keywords:** granulosa cell tumor, recurrence, FOXL2, array comparative genomic hybridization, chromosome instability

## Abstract

Introduction: Adult granulosa cell tumors (aGCTs) are extremely rare tumors characterized by the presence of the single missense mutation (c.402 C>G, p. C134W) in the *FOXL2* gene. These tumors are frequently associated with a slow, indolent disease progression and a high probability of aggressive tumor recurrence. Hence, the identification of molecular markers that are predictive of recurrence and/or aggressive behavior would be a great asset in the management of aGCT. The present study focused on the influence of the *FOXL2* genotype (heterozygous or homozygous) and copy number variations (CNVs) in recurrence by comparing the primary tumor with recurrent lesions in the same patient. We performed array comparative genomic hybridization (CGH) experiments and *FOXL2* genotyping by allelic discrimination on 40 tumor samples. Results and Discussion: In array CGH results of recurrent tumors, few samples presented the multiple chromosome losses and gains characteristic of chromosome instability (CIN). We also observed that three recurrent tumors and one primary tumor appeared to be homozygous for the *FOXL2* c.402C>G mutation. Interestingly, the homozygous *FOXL2* genotype was correlated with a shorter time to relapse. A change in the *FOXL2* genotype in cases of recurrence was correlated with the appearance of CIN. Conclusion: Despite the small number of matching primary and recurrent tumors analyzed here, the present study is the first to have shown that the *FOXL2* homozygous genotype and CIN are prevalent in recurrent aGCTs. The two mechanisms are probably linked, and both almost certainly have a role in the molecular transformation of aGCTs.

## INTRODUCTION

Granulosa cell tumors (GCTs) of the ovary are very rare, and account for less than 5% of all ovarian malignancies [1, 2]. Bryk *et al.* found that the incidence of granulosa cell tumors was very low - 0.6 to 0.8 per 100000 for their study period [3]. These tumors can be further divided into two distinct subtypes (juvenile GCTs and adult GCTs (aGCTs)) on the basis of their histologic, biochemical and molecular features. The primary molecular feature of aGCTs is the presence of a single pathognomonic missense mutation (c.402 C>G, p. C134W) in the *FOXL2* gene (coding for the forkhead box L2 transcription factor); this mutation is found in 94% to 97% of aGCTs [2, 4, 5], and is mainly observed in the heterozygous state. Most aGCTs are diagnosed early stage, so the prognosis is generally better than epithelial ovarian tumors; the 5-year survival rate is greater than 90% [6]. Adult GCTs are commonly associated with slow, indolent disease progression, a high recurrence rate, and aggressive recurrence. If surgery is no longer relevant, there are few other treatment options: the mortality rate among patients with advanced-stage or recurrent tumors is approximately 80% [7].

Although late recurrence is relatively common, the molecular mechanisms that underlie relapse or aggressive disease have yet to be identified. The pervasive somatic *FOXL2* mutation is likely to have a crucial role in the pathogenesis of aGCTs, and the mutation’s function in this setting has been extensively explored. However, the molecular consequences of the p. C134W mutation have not been elucidated. Given that a heterozygous *FOXL2* c.402 C>G mutation is present in almost all aGCTs, it is likely that other genetic changes are involved in the oncogenesis of aGCT. Recently, Fashedemi *et al.* reported on five cases of aGCT with uncommon areas of high-grade morphology. The researchers showed that the *TP53* mutation is likely to be involved in high-grade transformation [8]. Molecular markers that could predict recurrence and/or aggressive disease would be a great asset in the management of aGCT. Furthermore, a better understanding of the pathogenesis of late-stage disease might prompt the development of targeted therapies.

Genomic imbalance is known to be involved in oncogenesis [9]. Studies of genomic imbalance in aGCT have been performed but involved small numbers of sample and focused essentially on primary tumors [10, 11].

In the present study of formalin-fixed, paraffin-embedded (FFPE) primary and recurrent aGCTs, we used array competitive genomic hybridization (CGH) and an allelic discrimination technique to study the putative correlation between the *FOXL2* genotype and chromosome imbalance. We found that homozygosity for the *FOXL2* mutation and/or the presence of chromosome instability (CIN) was predictive of early recurrence and aggressive tumor behavior.

## RESULTS

### Characteristics of the study population and tumors

The FFPE aGCT samples were selected as described in the Materials and Methods. The patients’ clinical characteristics are summarized in Tables 1 and 2. A total of 40 samples (23 primary tumors and 17 recurrent tumors) were obtained from 27 patients. Nineteen of the 27 patients had primary tumors only, 4 had recurrent tumors only, and 4 had both primary and recurrent tumors.

**Table 1:** Clinical characteristics of the patients with primary aGCT samples

Sample number	Age	FIGO stage	Size (cm)	Initial surgery	CHR. aberrations	GI	Recurrence	Follow-up (month)
1	32	IA	10 × 7 × 8	SO	-13q	1	NO	LFU
2	62	IA	3,5	HSO	-22q	1	NO	LFU
4	45	IA	9 × 7,5 × 3	SO	-22q	1	YES	RFS 47, OS 51
5	26	IA	6 × 14 × 15	SO	nc	nc	NO	LFU
6	25	IA	11 × 8 × 7	SO	nc	nc	NO	RFS 146
7	61	IA	9	HSO	+1q, +6p, -22q	3	NO	RFS 50
8	55	I	5 × 4	SO	None	0	NO	LFU
9	34	IA	7 × 5 × 4	SO	None	0	NO	RFS 60, LFU
10	50	IA	13 × 10 × 5	SO	None	0	NO	RFS 120, LFU
12	63	IA	11 × 8 × 5	HSO	None	0	NO	LFU
16	34	IC3	Missing data	SO	None	0	NO	RFS 96, LFU
17	48	IA	7 × 6 × 6	SO	+14, -22q	2	YES	RFS 180, OS 204
18	47	IA	12 × 9	SO	-22q	1	NO	RFS 72, LFU
19	66	IA	16 × 12 × 10	HSO	nc	nc	YES	RFS 60
25	64	IC3	20 × 19 × 15	HSO	+19, -9p21	1	YES	RFS 36, OS 108
27	63	IA	7 × 6 × 7	HSO	+12	1	NO	RFS 72
28	31	IA	5,5	SO	+20, -1, -4, -5, -9, -11, -13, -14	37	NO	RFS 72
31	68	IA	7	SO	None	0	NO	RFS 12, LFU
32	74	IC1	6	SO	+14, -22	2	NO	RFS 36
33	41	IC1	5	HSO	-1q32.1-q12	1	NO	RFS 48
34	47	IA	23,5 × 20	HSO	nc	nc	NO	RFS 96
35	49	IA	24 × 23	HSO	+1q, -16q	2	NO	LFU
36	58	IA	5	HSO	None	0	NO	LFU

Primary + recurrent tumor pairs are indicated in color. SO: salpingo-oophorectomy; HSO: hysterectomy with bilateral salpingo-oophorectomy; CHR: chromosome; NC: not contributory; GI: genomic index; RFS: recurrence-free survival; OS: overall survival; LFU: loss to follow-up.

**Table 2:** Clinical characteristics of the patients with recurrent aGCT samples

Sample number	Age	Site of recurrence	Time interval (months)	CHR. Aberrations	GI
3	71	abdomen	60	-22q	1
11	67	abdomen	35	+8, +13, -2, -5, -11,-15,-18,-22	21
14	73	abdomen	84	+1q, +6, +10, +12 +20, -3q, -22q	17
20	76	missing data	missing data	None	0
22	75	missing data	missing data	nc	nc
23	48	abdomen	47	-22q	1
26	63	pelvis, abdomen	176	+14, -22q	2
30	74	abdomen	96	+1q,	1
38	45	abdomen	84	None	0
39	74	lung	9	nc	nc
40	79	abdomen	276	+1, +12, -21, -22	12
41	80	abdomen	288	+1q, +12, -19q, -21, -22	10
42	78	abdomen	264	+1q, +12, -21, -22	9
43	77	retroperitoneal area	252	+1q, +12, -21, -22	9
44	77	retroperitoneal area	252	+1, +12, -21, -22	12
45	73	abdomen	204	+12, -21, -22	8
46	73	abdomen	204	+12, -21, -22	8

The time interval (in months) between initial surgery and recurrence is given and primary + recurrent tumor pairs are indicated in color. CHR: chromosome; NC: not contributory; GI: genomic index.

On the 23 patients with primary tumors, 12 (52%) were aged under 50. All 23 primary tumors have been rated as International Federation of Gynecology and Obstetrics (FIGO) stage I. Only 10 (43%) of the 23 patients underwent the hysterectomy with bilateral salpingo-oophorectomy that is recommended in cases of early-stage rare ovarian tumors. For the 8 patients with recurrent tumors, the site of recurrence was the abdomen, and only one patient had a recurrent tumor outside the abdomen (in the lung). The median (range) time to relapse was 60 months (9–204).

### The FOXL2 genotype in the aGCT samples

**Figure 1:**
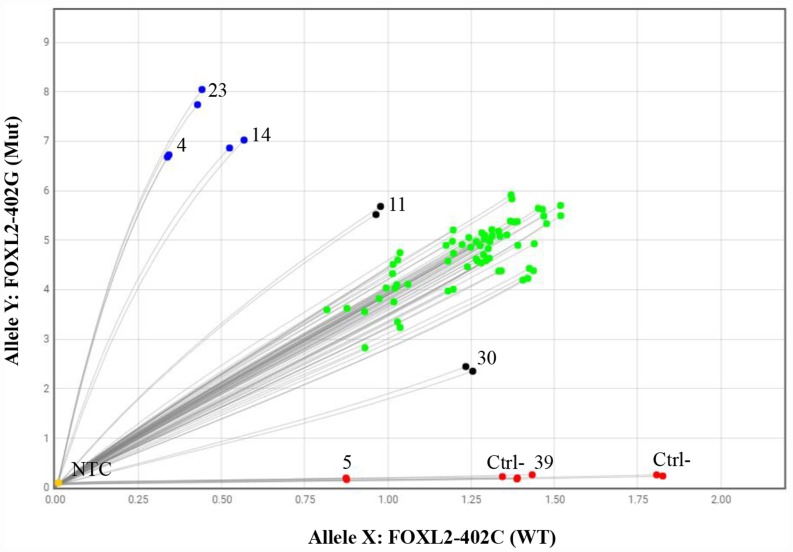
The qPCR allelic discrimination assay. Green dots represent samples that were heterozygous for the *FOXL2* c.402C>G mutation (with a fluorescent signal from both wild-type and mutant probes), blue dots represent samples that were homozygous for the *FOXL2* c.402C>G mutation (with a fluorescent signal from the mutant probe only), red dots represent wild-type samples (with a fluorescent signal from the wild-type probe only), black dots represent samples for which the genotype could not be determined, and yellow dots are the no-template controls. Each sample was tested in duplicate. *Ctrl-: negative control; NTCs: no-template controls.*

Thirty-eight (95%) of the 40 aGCT samples were positive for the *FOXL2* c.402C>G mutation in a qPCR allelic discrimination assay (Figure 1). One sample had a wild-type genotype (#39), and useful qPCR data were lacking for one sample (#5). Most of mutations were heterozygous, with a mutant allele population of approximately 50% (the green dots in Figure 1). Non-heterozygous alleles were found in five cases: three cases (#4, #14 and #23) clearly showed a mutated homozygous genotype (blue dots), one showed a high mutant allele frequency (case #11), and one showed a low mutant allele frequency (case #30). For samples #11 and #30, the tumor cell content (respectively 25% and 5% in a histologic assessment) was lower than in all the other samples tested (~90%). The low proportion of tumor cells in these two samples probably explains why the software could not reliably determine the genotype status (black dots). These results rather suggest that the correct genotype was a homozygous mutation for case #11 and #30.

**Figure 2:**
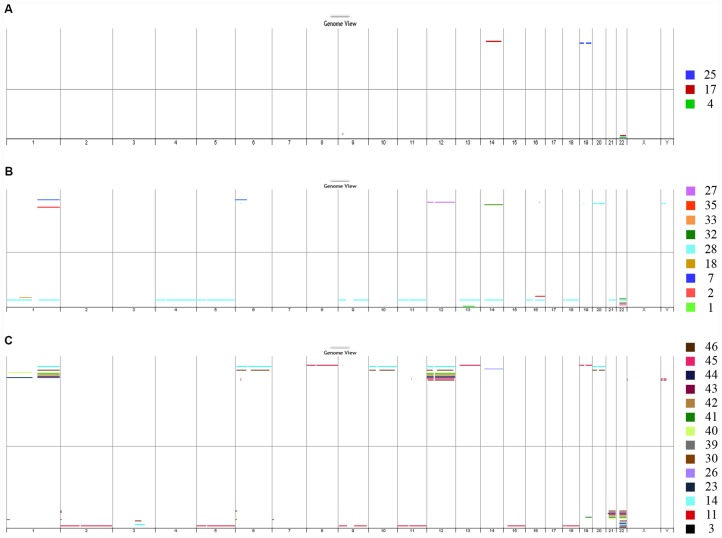
DNA copy number analysis. The genome view generated from array CGH data showed chromosomal aberrations in the DNA extracted from FFPE aGCT samples from recurrent primary tumors (**A**), non-recurrent primary tumors (**B**) and recurrent tumors (**C**). *CGH: comparative genomic hybridization; aGCT: adult granulosa cell tumor.*

**Figure 3:**
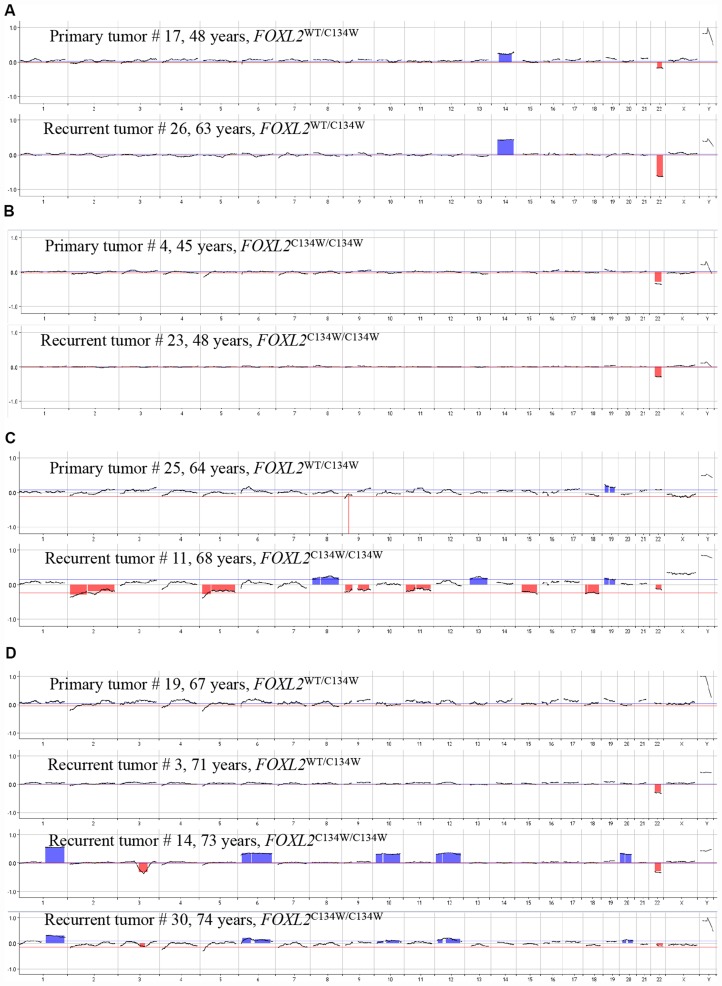
Changes over time in the *FOXL2* genotype and CNVs in pairs of matching primary and recurrent aGCTs. *Pair #1* (**A**)*; pair #2* (**B**)*; pair #3* (**C**)*; pair #4* (**D**)*.*

For all cases with homozygous mutations (#4, #11, #14 and #23), array CGH experiments did not show any chromosome 3 losses overlapping with the *FOXL2* gene region (Figure 3).

### Genomic copy number variations (CNVs) in aGCT samples

Four (17%) of the 23 primary tumor samples did not yield useful array CGH data, seven samples (30%) had no detectable aberrations, and 12 samples (52%) presented chromosomal imbalance (Figure 2A and 2B). When comparing primary tumors that recurred with primary tumors that did not recur, no distinctive patterns were observed. Interestingly, one primary tumor sample (case #28, shown in light blue in panel B) showed a complex array CGH profile with multiple chromosome gains and losses. The most frequent imbalance was loss of chromosome 22q (7 out of 12, 58%).

Concerning the 17 recurrent tumor sample, three (18%) had no detectable aberrations and 14 (82%) presented chromosomal imbalance: one or two chromosomal imbalance for cases #3, #23, #26, #30, and #39, and multiple chromosome gains and losses for cases #11, #14, #30, and #40-46 (Figure 2C). As observed for the primary tumors, the most frequent imbalance in recurrent tumors was the loss of chromosome 22q (12 out of 14, 86%).

The main difference between primary and recurrent samples was the higher frequency of chromosomal imbalance in recurrent tumors (14 out of 17, 82%), relative to primary tumors (12 out of 23, 52%). This was also reflected by the genomic index (GI). The highest values were seen in recurrent samples; the median (range) GI was 8.5 (1–21) for recurrent tumors, 1 (0-3) for primary tumors that did not recur and respectively 1, 2, not contributory and 1 for cases #4, #17, #19 and #25 (relapse).

### Morphology of aGCT samples

When comparing the histology of recurrent primary tumors with that of non-recurrent tumors, we did not observe any major differences. Similarly, primary tumors with and without loss of chromosome 22q did not have distinctive morphologies. Interestingly, the mitotic count per 10 high-power fields was much higher in tumor samples that were homozygous for the *FOXL2* c.402C>G mutation than in heterozygous aGCT samples. Lastly, tumor samples with multiple chromosome gains and losses exhibited marked nuclear atypia.

### *FOXL2* status and CNVs in primary vs. recurrent aGCTs

To evaluate the role of the *FOXL2* mutation and CNVs in the mechanism of recurrence, we compared primary tumor with the matching recurrent and metastatic lesions from our 4 patients (Figure 3).

For the primary and recurrent tumors in pair 1 (Figure 3A, cases #17 and #26; time to relapse: 15 years), the array CGH experiments revealed identical chromosome profiles (i. e. loss of chr 22q and gain of chr 14) and the same heterozygous mutant *FOXL2* genotype. For pair 2 (Figure 3B, cases #4 and #23; time to relapse: 3 years), the primary and recurrent tumors had the same chromosome profile (loss of chr 22q) and the same homozygous *FOXL2* mutation status.

Interestingly for pair 3 (Figure 3C, cases #25 and #11; time to relapse: 4 years), the primary tumor had a heterozygous mutant *FOXL2* genotype and no detectable chromosomal aberrations, whereas the recurrent tumor presented multiple chromosomal gains and losses and a homozygous mutant *FOXL2* genotype. The array CGH experiments did not show any loss on chromosome 3 overlapping the *FOXL2* gene region, which suggests that duplication of the mutant allele was combined with loss of the wild type allele (rather than loss of heterozygosity for *FOXL2*). Similar data were obtained for pair 4 (cases #19, #3, #14 and #30, Figure 3D). Although the primary tumor did not show any chromosomal aberrations, the first recurrence (case #3, 4 years after the initial diagnosis) presented loss of chr 22q. The subsequent recurrences (case 14, 6 years after the initial diagnosis, and case #30, 7 years after the initial diagnosis) presented multiple gains and losses. Interestingly, the acquisition of these CNVs was associated with a homozygous mutant *FOXL2* genotype, as observed in the recurrent tumor from pair 3.

## DISCUSSION

In 2009, Shah *et al*. reported that the somatic c.402C>G missense mutation in *FOXL2* was present in 97% of aGCTs, which strongly suggested a direct causal role [4]. Since then, several research groups have unsuccessfully sought to understand the mechanistic role of this mutation on the recurrence of aGCT [12, 13]. In fact, the rarity and clinical course of aGCT (characterized by indolent growth with late recurrence) have made it difficult to analyze large numbers of patients and even harder to compare primary and recurrent tumors from the same patient. The objective of the present study was therefore to determine respective roles of the *FOXL2* genotype (heterozygous or homozygous) and CNVs in the mechanism of late/aggressive aGCT recurrence by comparing primary and recurrent tumors.

### Chromosome instability in recurrent aGCTs

An array CGH analysis of FFPE samples of 40 surgically resected aGCTs confirmed that the most frequent CNV was loss of chromosome 22q; this had occurred in 52% of the primary tumors and 82% of the recurrent tumors. Similar values (ranging from 30 to 53%) have been reported in the literature [10, 11, 14, 15].

There are also literature reports of trisomy 12, trisomy 14, and loss of chromosome 16, although these CNVs were less frequent in our study. This discrepancy might be due (at least in part) to the number of samples used in the literature studies (10, 15, and 21 samples in references [10, 11, 14], respectively) or by the high proportion of recurrent tumors in our series [15]. When calculating the GI for each sample, we could not determine a predictive cut-off. Firstly, few primary tumor recurred. Secondly, the GI of recurrent primary tumors and that of non-recurrent primary tumors both ranged from 0 to 3.

Considering that aGCTs frequently show allelic deletions of chromosome 22q, our results suggest that the inactivation of one or more tumor suppressor genes in this region is important for tumorigenesis. Potential tumor suppressor candidate genes have been identified in this region. For instance, the somatic loss or inactivation of the *NF2* gene was shown to be frequently associated with the development of isolated nervous system tumors and mesotheliomas [16]. The *SMARCB1/INI1* gene was identified as a potent tumor suppressor in rhabdoid tumors, epithelioid sarcomas, schwannomatosis, synovial sarcomas, and other conditions [17]*.* Recently, Pang *et al.* reported recurrent genomic inactivated *DEPDC5* mutations in GISTs; the mutations were prognostic in that they were associated with aggressive GISTs, GIST progression, and low sensitivity to KIT inhibitors [18]. The loss of tumor suppressor genes in 22q region has yet to be reported in aGCTs.

The array CGH results were of great value for the assessment of recurrent tumors, few of which (cases #11, #14, #30 and #40-46) presented multiple chromosome losses and gains (Figures 2 and 3). In fact, the recurrent tumors’ array CGH profiles were characteristic of somatically acquired aneuploidy. Aneuploidy reflects both whole-chromosomes gains or losses and non-balanced chromosome rearrangements, including deletions, amplifications, and translocations of large regions of the genome [9]. The presence of structural chromosome aberrations and CNVs often reflects ongoing CIN (a type of genomic instability, along with microsatellite instability (MSI) and nucleotide instability). Genomic instability is a hallmark of cancer and leads to an increase in genetic alterations; in turn, this enables the acquisition of additional defects required for tumorigenesis and progression. A high degree of CIN has been associated with a poor clinical outcome in several types of cancer [19, 20].

The oncogenic mechanism of CIN has been particularly well described in colorectal cancer, where tumors with CIN do not display MSI [21]. Our observation of aneuploidy mainly in recurrent tumors suggests that CIN may be involved in the mechanism of recurrence of aGCTs. All aGCT samples were tested for MSI (data not shown), and all displayed a stable microsatellite phenotype - suggesting that CIN is the preferred pathway in these tumors.

Aneuploidy was detected in only one primary tumor (case #28, Figure 2B). Interestingly, this patient was diagnosed at the age of 31, which is quite early for aGCTs; the median age at presentation is reportedly 50–54 [22]. This patient has been followed up (a physical examination, lab tests, a computerized tomography (CT) scan and pelvic magnetic resonance imaging (MRI)) since surgery, and shows no signs of recurrent disease. Furthermore, a histopathologic assessment revealed a typical architecture but marked nuclear atypia for size and shape, which is unusual for these indolent tumors. Again, these data strongly suggest that CIN is involved in the oncogenetic process in aGCTs. As recently showed by Fashedemi *et al.*, p53 was found to be expressed at a low level by 90% of the tumor cells in this single case - further suggesting a role for p53 in carcinogenesis.

The presence of chromosomal alterations in a tumor does not necessarily indicate that instability will persist, and further investigation is usually required. *In vitro* analyses of mitotic abnormalities (including lagging chromosomes and multipolar mitoses) and chromosome gains and losses of chromosomes during cell division would be needed to confirm the persistence of CIN in aGCTs [23]. Given that measuring CIN through the rate of acquisition of chromosomal changes is difficult in solid tumors, the CNV burden can be used as a surrogate marker. Moreover, the data obtained here for pair 4 and for samples #40-46 suggest that chromosomes alterations persist over time.

### The homozygous *FOXL2* genotype in recurrent aGCTs

Our results showed that the *FOXL2* c.402C>G mutation was present in 38 of the 40 morphologically identified aGCTs (95%); this is in line with the literature data [4, 10, 11, 24, 25]. We observed a heterozygous phenotype for most of the samples but also found that three recurrent tumors (cases #23, #11 and #14) and one primary tumor (#4) appeared to be hemizygous or homozygous for the c.402C>G mutation. Since the array CGH experiments did not show any loss on chromosome 3 overlapping the *FOXL2* gene region, these four aGCT samples were more likely to be homozygous than hemizygous. Moreover, the C134W/C134W mutation was not present in the primary tumors but appeared in the recurrent tumor (case #11) in pair 3 and in the late recurrent event (case #14) for pair 4 - suggesting that the acquisition of a homozygous *FOXL2* genotype was a secondary event in these cases. Also, the change in the *FOXL2* genotype (from heterozygous to homozygous) in the recurrent tumor was correlated with the appearance of CIN in pair 3 and 4. In contrast, the primary tumor in pair 2 (case #4) presented the homozygous genotype. Overall, we observed the persistence or acquisition of the homozygous *FOXL2* genotype in 3 of the 4 pairs.

Interestingly, the observation of the homozygous *FOXL2* genotype was correlated with a short time to relapse (2 and 1 years for pair 4, 3 years for pair 3, and 3 years for pair 2) relative to the literature studies in which the median disease-free survival time is between 5 and 10 years [15, 22–24]. In contrast, both the primary and recurrent tumors for pair 1 had a heterozygous genotype, and the time to relapse was longer (15 years). Only one recurrent sample (case #39) with a short time to relapse had not the *FOXL2* c.402C>G mutation (wild-type genotype). This recurrent tumor might have be homozygous for another *FOXL2* mutation. Although other somatic *FOXL2* variants are rare, a number have been identified [4, 26]. In contrast, this exceptionally early recurrence might have resulted from incomplete surgery at the start of the course of disease or from other genomic/chromosomic abnormalities.

Taken as a whole, these data strongly suggest that the acquisition of *FOXL2* homozygous genotype is likely to be involved in tumor recurrence, and might be a marker of early recurrence. However, these data need to be confirm in a larger series of matching pairs of primary and recurrent tumors.

Although homozygous *FOXL2* c.402C>G mutation status is rare, it has been reported before (3 out of 89 patients in one study, and 2 out of 21 in another [4, 11]). As in our cases, Geierbasch *et al*. found that two mechanisms (loss of the wild type allele alone, or duplication of the mutant allele plus loss of the wild-type allele) were involved. However, the present study is the first to have found a change in *FOXL2* mutation status between the primary tumor and the recurrent tumor. This finding supports the hypothesis whereby *FOXL2* can act as a tumor suppressor gene rather than as an activating mutation or a gain-of-function mutation.

Carcinogenesis is a multistep process that can arise from a combination of mutations in oncogenes or tumor suppressor genes or from epigenetic changes in DNA. It is not yet known which of these processes are involved in aGCT, although recent genetic studies of primary and recurrent tumors have identified mutations in the promoter of the *TERT* gene (coding for telomerase reverse transcriptase), in the *KMT2D* gene (coding for a histone lysine methyltransferase) and in the *TP53* gene [8, 14, 26]. These mutations’ additional contributions to carcinogenesis (along with the *FOXL2* mutations and CIN notably described here) have yet to be determined.

## MATERIALS AND METHODS

### Patients and samples

The study’s objectives and procedures were approved by the local investigational review board (*Commission Interne d’Evaluation des Projets de Recherche Hors Loi Jardé, Amiens, France*). Clinical data from 27 patients having undergone surgical excision of aGCTs at Amiens-Picardie University Medical Center and other hospitals (CH Beauvais, CHU Reims, and CH St-Quentin) between 1999 and 2017 were included in the present analysis. Formalin- fixed paraffin-embedded samples from primary and/or recurrent tumors were collected from these patients. Tumors were staged in accordance with the FIGO system (2014) [27].

### Extraction of genomic DNA

Genomic DNA was obtained from FFPE tissue by automated extraction with the Tissue Preparation System (Siemens) and the VERSANT^®^ Tissue Preparation Reagents kit. The DNA concentration was determined using the Qubit dsDNA HS Assay Kit (ThermoFisher).

### The qPCR allelic discrimination assay

A custom TaqMan qPCR single-nucleotide polymorphism genotyping assay (Life Technologies) was performed according to the manufacturer’s instructions. The *FOXL2* c.402C>G mutation was genotyped as described previously [4], using the following primers: 5′-GCGCAAGGGCAACTACTG-3′ (forward) and 5′-CGGTAGTTGCCCTTCTCGAA-3′ (reverse), along with a wild-type specific probe (5′-FAM dye- CATGTCTTCCCAGGCCG- non-fluorescent quencher (NFQ)) and a mutation-specific probe (5′-VIC dye-CATGTCTTCGCAGGCCG-NFQ) included in the genotyping master mix. Reactions were run on a QuantStudio 7 Flex Real-Time PCR System (Life Technologies). A reaction volume of 5 μl was used for each replicate well; it included 2.5 μl of a 2× TaqMan Genotyping master mix (Life Technologies), 0.125 μl of a 40× custom synthesized allelic discrimination primer/probe mix (Life Technologies), 1 μl of DNA suspension (standardized to contain 15 ng), and water. After denaturation at 95° C for 10 minutes, DNA was amplified over 40 cycles (95° C for 15 seconds, and 60° C for 1 minute). DNA from molecularly and histologically confirmed aGCTs was used as a positive control, and DNA from juvenile GCT samples was used as a negative control. Water was used as a no-template control. Each sample was tested in duplicate.

### DNA copy number analysis

Array CGH experiments were performed according to the manufacturer’s instructions (Agilent Technologies) after optimization for DNA obtained from FFPE tissue samples. Briefly, 500 ng of FFPE DNA labeled with Cy3-dCTP or Cy5-dCTP was competitively hybridized with heat-fragmented female reference genomic DNA on 8 × 60 K CGH microarrays (Sureprint G3 Human). The arrays were washed and scanned, and the images were obtained using Feature Extraction software (Agilent Technologies). The scanned data were analyzed with Cytogenomics software (version 4.0, Agilent Technologies). The ADM-2 algorithm and a threshold value of 6.0 were applied, along with appropriate filters. Gains and deletions of chromosomal regions were considered when (i) the corresponding plotted oligoprobes presented an absolute log ratio ≥ 0.25, and (ii) the minimum size of region for a gain/deletion was ≥ 1000 kb. Gene amplification was considered when the plotted oligoprobes targeting the gene had a log ratio ≥ 0.25.

The genomic index was calculated for each profile as follows: genomic index = *A^2^/C*, where *A* is the total number of alterations (segmental gains and losses), and *C* is the number of chromosomes involved.

## CONCLUSIONS

Although we studied a small number of matching primary and recurrent aGCTs, we observe a homozygous *FOXL2* genotype and CIN mainly in recurrent tumors rather than primary tumor. We also observed that a homozygous *FOXL2* genotype and/or the presence of CIN appeared to predict early recurrence and aggressive tumor behavior. More primary + recurrent tumor pairs would be necessary to confirm these data but are very difficult to obtain. The two mechanisms (*FOXL2* mutations and CIN) are probably interrelated and definitely have a role in the molecular transformation of aGCTs.

## Abbreviations

aCGH: array comparative genomic hybridization
aGCT: adult granulosa cell tumor
CNV: copy number variation
FFPE: formalin-fixed, paraffin-embedded
FOXL2: forkhead box L2
CIN: chromosome instability
FIGO: International Federation of Gynecology and Obstetrics
MSI: microsatellite instability
